# MANTRA: Improving Knowledge of Maternal Health, Neonatal Health, and Geohazards in Women in Rural Nepal Using a Mobile Serious Game

**DOI:** 10.3389/fpubh.2020.584375

**Published:** 2020-12-11

**Authors:** Sonja Mueller, Delphine Soriano, Andrei Boscor, Naomi M. Saville, Abriti Arjyal, Sushil Baral, Maureen Fordham, Gareth Hearn, Rachya Kayastha, Patty Kostkova

**Affiliations:** ^1^Institute for Risk and Disaster Reduction, University College London, London, United Kingdom; ^2^Institute for Risk and Disaster Reduction Centre for Digital Public Health in Emergencies (dPHE), University College London, London, United Kingdom; ^3^Institute for Global Health, University College London, London, United Kingdom; ^4^Health Research and Social Development Forum, Kathmandu, Nepal; ^5^Institute for Risk and Disaster Reduction Centre for Gender and Disaster, University College London, London, United Kingdom; ^6^Hearn GeoServe Ltd., Worthing, United Kingdom

**Keywords:** user evaluation, serious mobile game, digital health intervention, interdisciplinary research, knowledge assessment, mobile learning, educational game, serious game

## Abstract

Serious games, conveying educational knowledge rather than merely entertainment, are a rapidly expanding research domain for cutting-edge educational technology. Digital interventions like serious games are great opportunities to overcome challenges in low-and-middle-income countries that limit access to health information, such as social barriers like low-literacy and gender. MANTRA: Increasing maternal and child health resilience before, during and after disasters using mobile technology in Nepal takes on these challenges with a novel digital health intervention; a serious mobile game aimed at vulnerable low-literacy female audiences in rural Nepal. The serious game teaches 28 learning objectives of danger signs in geohazards, maternal, and neonatal health to improve knowledge and self-assessment of common conditions and risks to inform healthcare-seeking behavior. Evaluations consisted of recruiting 35 end users to participate in a pre-test assessment, playing the game, post-test assessment, and focus groups to elicit qualitative feedback. Assessments analyzed knowledge gain in two ways; by learning objective with McNemar tests for each learning objective, and by participant scores with paired *t*-tests of overall scores and by module. Results of assessments of knowledge gain by learning objective (McNemar tests) indicate participants had sufficient prior knowledge to correctly interpret and respond to 26% of pictograms (coded AA), which is a desirable result although without the possibility of improvement through the intervention. The geohazard module had greatest impact as 16% of responses showed knowledge gain (coded BA). The two most successful learning objectives showing statistically significant positive change were evidence of rockfalls and small cracks in the ground (*p* = < 0.05). Assessment of knowledge gain by participant scores (paired *t*-tests) showed the 35 participants averaged a 7.7 point improvement (*p* < 0.001) in the assessment (28 learning objectives). Average change in knowledge of subdivided module scores (each module normalized to 100 points for comparison) was greatest in the geohazard module (9.5 points, *p* < 0.001), then maternal health (7.4 points, *p* = 0.0067), and neonatal health (6.0 points, *p* = 0.013). This evaluation demonstrated that carefully designed digital health interventions with pictograms co-authored by experts and users can teach complex health and geohazard situations. Significant knowledge gain was demonstrated for several learning objectives while those with non-significant or negative change will be re-designed to effectively convey information.

## Introduction

Mobile phones and mobile health (mHealth) initiatives have demonstrated value for overcoming obstacles of remote populations, rough terrain, and limited resources to distribute important public health information (1–4). Serious games, which are games with an educational purpose beyond entertainment, are an important, as yet underutilized mHealth opportunity in low and middle income (LMIC) settings (5, 6).

The “Maternal and Neonatal Technologies in Rural Areas (MANTRA): Increasing maternal and child health resilience before during and after disasters using mobile technology in Nepal” project investigated building women's resilience by improving access to information and communications before, during, and after environmental disasters by developing mobile technology to support and expand existing participatory learning public health interventions and social protection mechanisms.

Within this research aim and context, we investigate knowledge gain amongst participants from playing a serious game intervention designed by the research team. Analyzing knowledge change highlighted successes and improvements within the MANTRA serious game to inform the next development phase, as well as insights that are transferable to similar research projects.

This research builds on the potential for mHealth interventions in LMICs to reach a low literacy audience, serious games as a tool for conveying educational messages, and rapidly increasing accessibility of digital technology and supporting infrastructure in low income settings.

Evaluating learning through knowledge assessment is essential to quantify an educational experience. Traditionally in serious games, pre- and post-play assessments of knowledge take place outside the game by a test or a survey, in line with teaching and intervention assessment methods (7–10). Available research reports mixed results of serious games delivering educational content (8, 11–13), while noting that usability, acceptability and cultural appropriateness impact learning (14–16).

Implementing mHealth initiatives in LMICs means reaching a target audience with variable levels of education such that illiteracy is an important design consideration (17). To include all members of the population, the MANTRA serious game is designed for an illiterate or low-literacy user. Lessons for developing serious games for low literacy users could be transferred from research aimed at other illiterate audiences, such as toddlers and young children. One example is a game consisting solely of images, animation, and audio, “Listening with Lemur,” for children aged 1.5 to 3 years with recent cochlear implants (18).

MHealth is a rapidly expanding area in healthcare systems and health education, and Kostkova succinctly presents the huge potential of mHealth to impact the health sector and future challenges facing the field (6). Governments, non-governmental organizations, and academics recognize the potential of mHealth to implement health campaigns at low cost (6, 19–21). Considerations for implementing an mHealth intervention in Nepal are highlighted by Style et al. (3), such as poor infrastructure limiting network access, variable electricity hindered device charging, and misuse of devices by family members (3). Continuing research into the needs, context, and existing health systems in a region or nation will ensure the technology is useful and accepted by patients, health workers, and healthcare systems (6, 21).

Nepal is a suitable study area for testing an mHealth intervention because of rising access to mobile phones, risks inherent to a dispersed population in a geologically active region, and vulnerabilities at national, community and individual scales. Communication links have been strengthened as the number of mobile phone subscriptions in Nepal reached 110 subscriptions per 100 population in 2016 (22), growing from about 9 million subscriptions in 2010 to over 32 million in 2016 (23). Nepal's Demographic and Health Survey 2016 reports that mobile phone ownership is highest among the 20–24 age group for both women and men, at 85 and 96%, respectively (24). Rapidly increasing access to mobile phones supports the use of mobile health tools to reach a population.

Literacy among Nepali women is one social barrier to accessing digital health interventions and a vital design consideration for the MANTRA serious game. In 2011, the World Bank reported Nepal's national literacy rate as 60% for adults over 15 years of age, and 85% in the age group 15–24 years. Compared to national rates, women have adult literacy rate of 49%, and a literacy rate of 80% within the 15–24 year age group (25). These lower literacy rates among women suggest there is a significant low-literacy population that would be difficult to reach through written information and could benefit from interventions designed to target low-literacy audiences.

In Nepal, healthcare is delivered by rural health workers at the local health post, with outreach conducted by female community health volunteers (FCHVs) to provide healthcare education in catchment areas each with ~1,000 people (26). In a disaster situation like the 2015 Kathmandu earthquake, broken transportation links isolated communities from healthcare and medical advice (27, 28). Kavrepalanchok district was one of 14 districts highly affected by the 2015 earthquake and yet accessible for research, so heavily earthquake-affected study locations were selected from this district, which now lies in Province 3 according to the new Federal State system (27, 28).

This setting in Nepal has increasing access to mobile phones, a hazardous landscape, unreliable transportation links, and basic rural health systems that feed into higher referral centers which are often difficult to reach, especially in emergencies. These characteristics makes it a suitable location to test a serious game for mobile phones focused on health and hazards.

## The Mantra Serious Game

**Figure 1:**
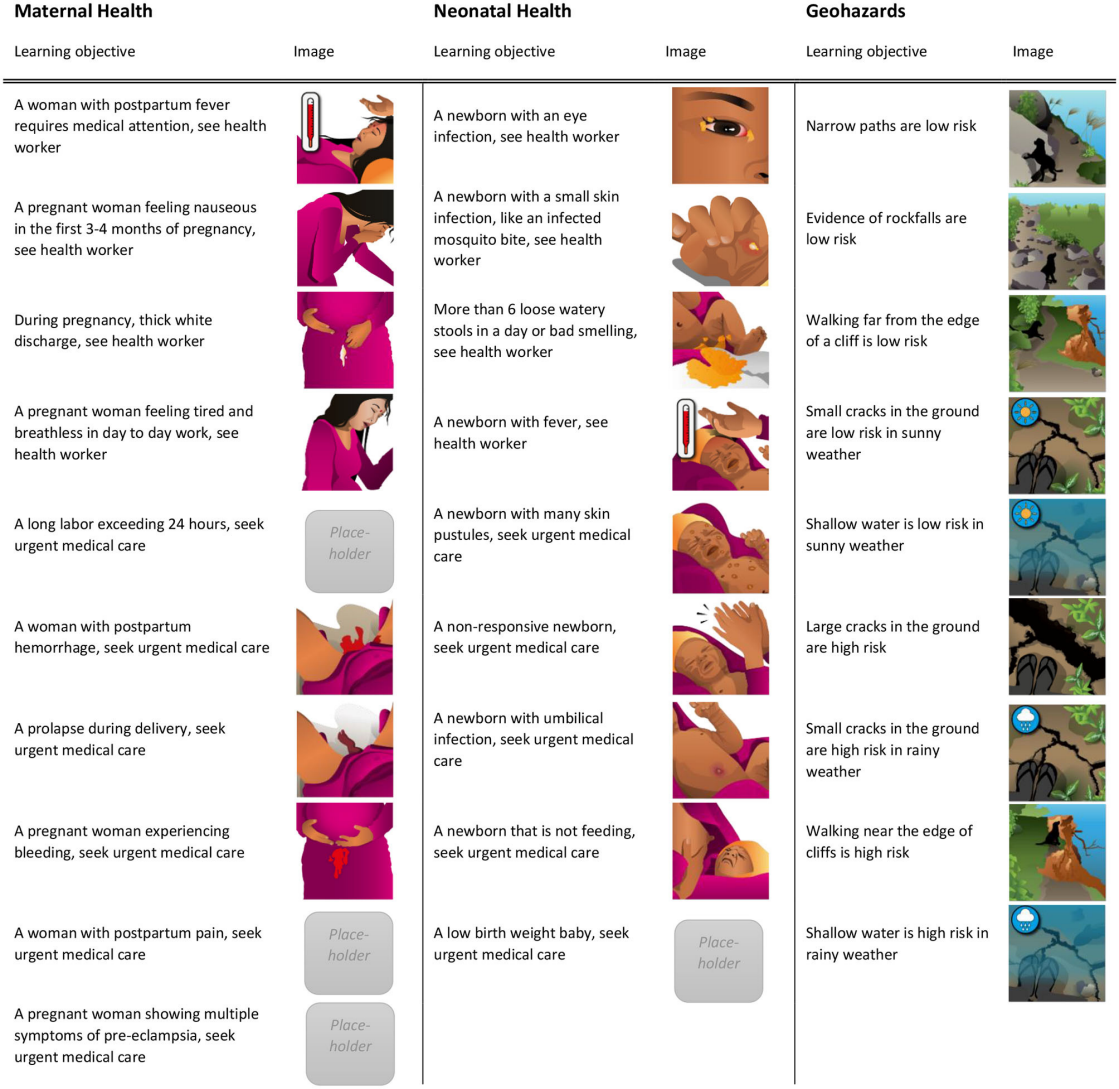
The learning objectives for the three modules are shown here with their corresponding artwork images as they appear in the game. Some of the images are placeholders modified from existing picture card interventions including MIRA's (31) perinatal women's groups intervention (32, 33), and some placeholder images are not presented here since they were removed from the game after the field tests for further development (infant with rapid breathing, infant with indrawn chest, and infant with convulsions). Reprinted from Mueller et al. (29).

The MANTRA serious game was built upon qualitative research conducted within the MANTRA project. As described elsewhere (29), key elements of the serious game are learning objectives, game mechanics, and design features. The mobile serious game covers three broad topics or modules; maternal health, neonatal health, and geohazards, each with 9 or 10 learning objectives. Since the game targets a low literacy audience, the design focused on visual communication with no text. Learning objectives were illustrated with pictograms co-created with researchers in Europe and Nepal. The pictogram design process is discussed in (30). The learning objectives are presented below with their corresponding pictograms in Figure 1.

**Figure 2:**
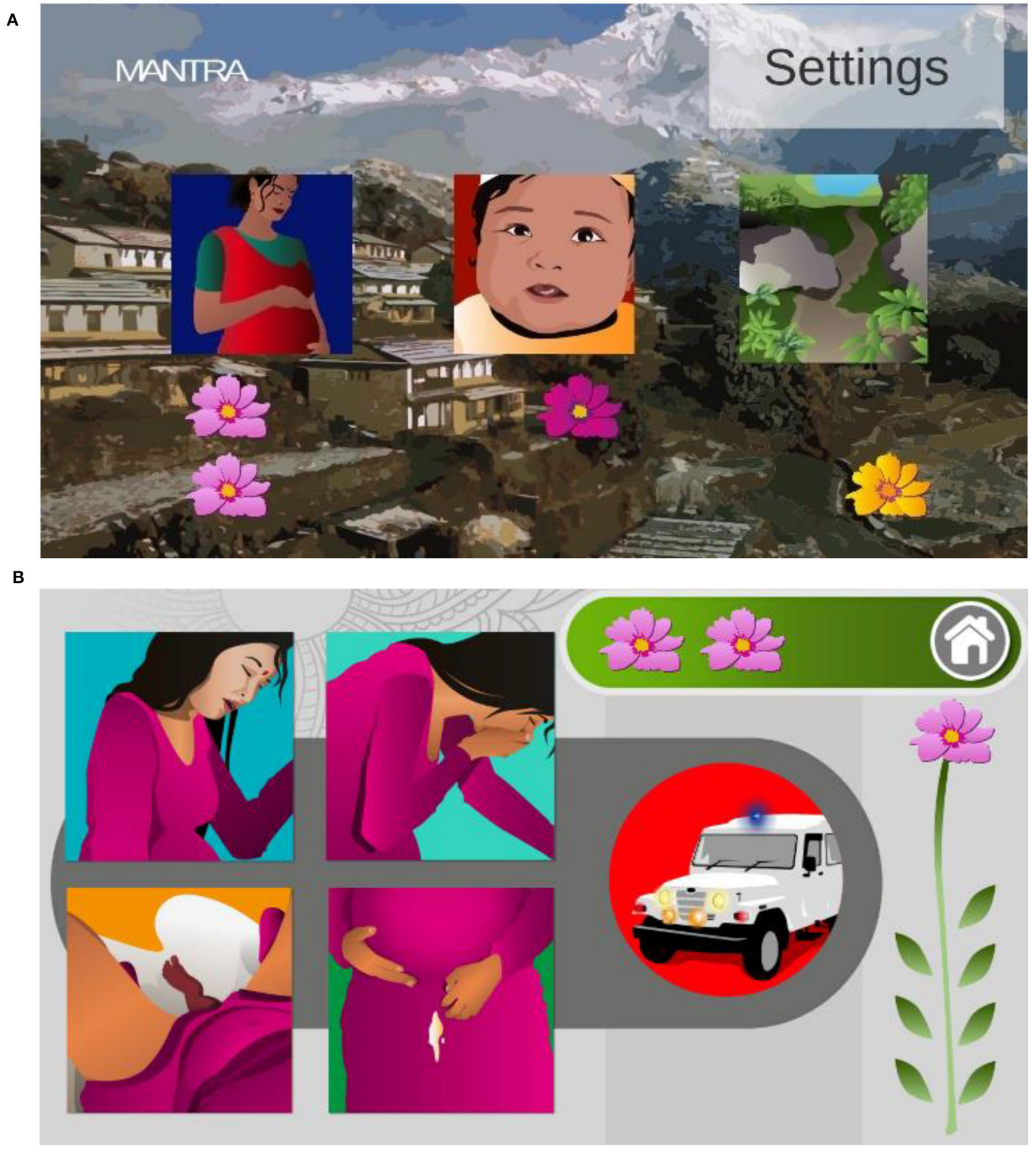
Screenshots from the mobile phone game. **(A)** The home screen showing three tiles that take the user to the corresponding module when clicked and flowers awarded for successfully completing the module. **(B)** A difficult question asking which of the four choices of situations requires an urgent response. Reprinted from Mueller et al. (29).

The home screen in Figure 2A contains three tiles corresponding to the three modules; maternal health, neonatal health, and geohazards (left to right). Each module contains three levels of difficulty and Figure 2B shows a question in the most difficult level with four possible answers.

**Figure 3:**
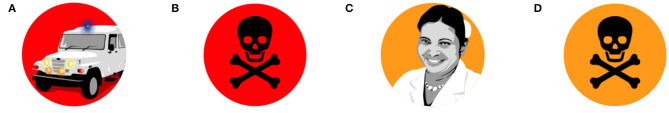
Pictograms **(A,B)** represent seeking urgent medical care and high risk, respectively. Pictograms **(C,D)** represent seeing a health worker and lower risk, respectively. Reprinted from (29).

A drag and drop interface kept the game mechanics simple as players match pictograms of conditions and risk levels. To play, participants interpret a given action or risk level from Figure 3, then interpret and choose one of the square situation images of learning objectives from Figure 1 and finally, drag and drop the chosen square onto the circle. The development process and considerations are presented in (29).

## Materials and Methods

We assessed knowledge gained from the serious game intervention using paired *t*-test and McNemar test methods, which are common for assessing impact of educational interventions (8, 9, 12, 13, 34).

### Study Locations and Design

Testing took place in Kavrepalanchok district and suburban Kathmandu, with suitable locations identified by colleagues at Health and Social Development Forum (HERD) on the basis of damage and visible geohazards arising from the 2015 earthquake. Field testing of the serious game took place in early November 2017, facilitated by HERD and Nepal-based colleagues. The game content was consistent as no major changes were made to the game during this set of tests.

### Field Test Format

**Figure 4:**
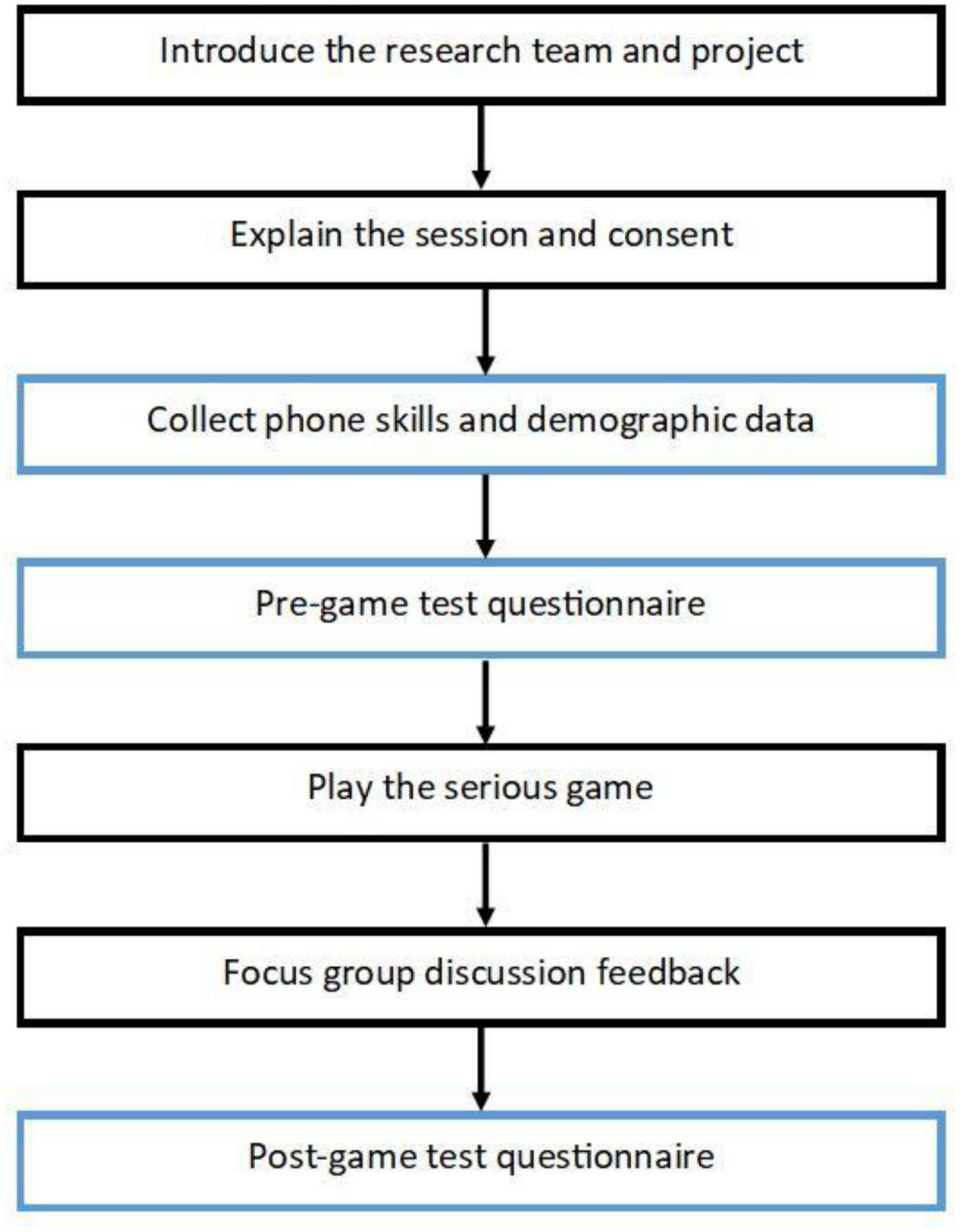
Visualization of the field test format with steps in chronological order. Blue boxes indicate the data collection steps that are the source of data for the statistical analyses presented in this paper. Focus group methodology and results are presented elsewhere [(35); Kayastha et al. in preparation]. Reprinted from Mueller et al. (29).

Thirty-five participants were recruited to the study for the November 2017 field tests. First, the project was verbally explained in Nepali, participants were given the opportunity to ask questions of the researchers, and informed written consent obtained. Second, participants provided data about demographics and experience in handling smartphones. A pre-game test questionnaire of the 28 learning objectives established a baseline for the knowledge assessment, followed by 10–30 min of individual game play, a focus group discussion [(35); Kayastha et al. in preparation] and concluding with an identical post-game test questionnaire of the learning objectives. Each learning objective was considered as one question. A facilitator administered the test questionnaires to the group as each participant marked answers on their own questionnaire paper. Paper questionnaires were used due to lack of computer facilities. Although participants were advised to play individually, laboratory testing conditions were not possible in open community spaces, so some discussed the game with others as they were playing. For field testing, the game was installed on six Samsung Galaxy 7 smartphones to minimize the chance of variation and unforeseen problems. Figure 4 visualizes the steps in the field testing process.

### Knowledge Assessment of Learning Objectives

Knowledge assessment data compiled from the pre- and post- game questionnaires were analyzed using several statistical methods. Paired pre- and post-game test responses of each participant were compiled and digitized for analysis in IBM SPSS 22 statistical package. Participant test scores were normalized to 100 points for straightforward comparison, such that in a test of 28 questions, a score of 100 represents 28 correct answers, while a score of zero represents 28 incorrect answers. The change of knowledge was calculated by subtracting the pre-game test scores from the post-game test scores, where a positive difference indicates a higher post-game test score, while a negative difference indicates a lower post-game test score.

A series of McNemar analyses looked at each learning objective question on its own. This statistical test analyzes categorized answers by correct and incorrect answer sequences for each learning objective and calculates a significance for change of each learning objective. Desirable and undesirable responses to each learning objective were counted to assess the success of each learning objective. The McNemar analyses are presented in Assessment of Knowledge Gain by Learning Objective.

A paired *T*-test assessed the significance of changes in knowledge assessment scores of all 35 participants. Four tests were conducted, one looking at 28 learning objectives overall, and tests for each of the three modules. The results of these four paired *T*-tests are presented in Assessment of Knowledge Gain by Module and Overall.

Participants were asked about smartphone ownership, gender, education, age, community roles (community women, FCHVs, community men), and location. Age groups of below 35 vs. 35 years and above were chosen to provide sufficient numbers for statistical analysis and coincide with national surveys like Demographic and Health Surveys. Numerical data from years of education were converted to categorical variables of no formal qualifications, completion of primary education, and completion of secondary education. Participant characteristics are presented in Demographics and Phone Use.

Relevant insights on learning effectiveness from game play observations and focus group discussions are briefly mentioned in section Key insights from observations and focus group discussions. [(35); Kayastha et al. in preparation] contains more detailed analysis of focus group discussions, and specifics of artwork are discussed in Soriano et al. (30).

### Ethics Approval and Consent to Participate

The MANTRA project research studies were approved by the University College London Ethics Committee in London, United Kingdom [10547/001] and the Nepal Health Research Council in Kathmandu, Nepal [Reg. No. 105/2017]. HERD co-authors and researchers explained the research to participants and all focus group and interview participants provided written informed consent. Participants were given low-value in-kind incentives to compensate for their time, such as a meal and tea. The funding body played no role beyond the funding call in the design of the study, data collection, analysis, data interpretation, or writing the manuscript.

## Results

By quantifying the impact of the serious game intervention on knowledge gain we aim to provide insight into the effectiveness of the content and delivery of the serious game. The various statistical tests applied to the data approach knowledge gain from several perspectives that will inform the next iteration of game design.

### Assessment of Knowledge Gain by Learning Objective

In a McNemar test, each participant response gets two letters, the first letter representing the answer on the pre-game test and the second letter corresponding to the answer on the post-game test. Correct responses are coded *A*, and incorrect responses are coded *B*, producing responses AA, AB, BA, and BB.

Desirable responses (AA, BA) are summarized in Table 1. The highest prior knowledge was found in the maternal health module, with 32% of responses coded AA and only 9% improvements coded BA. This is unsurprising as many of our respondent were FCHVs and women who had already had a baby, who were familiar with maternal health concepts from community health programs and personal experience. In contrast, the neonatal health module had the lowest percent of AA responses at 21% as well as more uniform results of improvement where 10% of responses were coded BA. These results were surprising as infant health is also covered in community programs. Geohazard learning objectives, like neonatal health, also showed a low prior knowledge response with only 25% coded as AA while 16% showed improvements coded BA.

**Table 1:** Summary of desirable responses to knowledge assessment of learning objectives.

	**Desirable responses**	**AA**		**BA**	
Total responses	38%	254	26%	114	12%
Responses for geohazard module	41%	78	25%	50	16%
Responses for maternal health module	41%	111	32%	32	9%
Responses for neonatal health module	31%	65	21%	32	10%

Looking at the McNemar tests in greater detail, coded response pairs in Tables 2–4 are useful to infer participant prior knowledge, interpretation of pictograms, and the importance of anecdotal experience in small communities.

**Table 2:** Summary of the maternal health module.

**Maternal health learning objectives**		**Response pairs** **(A = correct, B = incorrect)**				**McNemar statistics**	**Significance**	**Desired outcome** **(% of 35 question pairs)**			**Undesirable outcome** **(% of 35 question pairs)**		
	**Image**	**AA**	**AB**	**BA**	**BB**	***X*^**2**^**	***p***	**AA**	**BA**	**Combined**	**AB**	**BB**	**Combined**
A woman with postpartum fever requires medical attention, see health worker	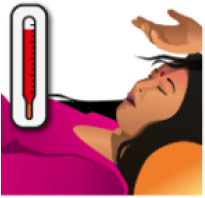	11	6	8	10	0.071	0.791	31%	23%	54%	17%	29%	46%
A pregnant woman feeling nauseous in the first 3–4 months of pregnancy is less urgent, see healthcare worker	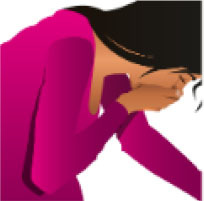	0	5	0	30	3.2	0.063	0%	0%	0%	14%	86%	100%
During pregnancy, thick white discharge is a minor danger. See health worker	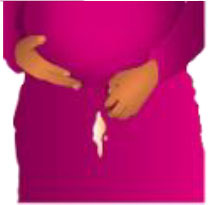	6	13	3	13	5.062	0.021^*^	17%	9%	26%	37%	37%	74%
A pregnant woman feeling tired and breathless in day to day work is less urgent, see health worker	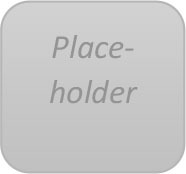	30	0	4	1	2.25	0.125	86%	11%	97%	0%	3%	3%
A long labor exceeding 24 h requires urgent medical care	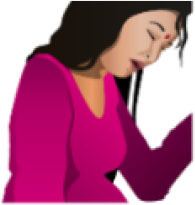	35	0	0	0	No Calc	No Calc	100%	0%	100%	0%	0%	0%
A woman with postpartum hemorrhage requires urgent medical care	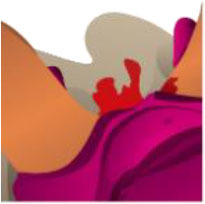	0	2	1	32	0	1	0%	3%	3%	6%	91%	97%
A prolapse during delivery requires urgent medical care	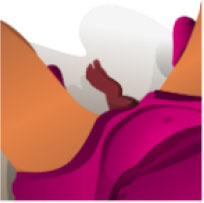	0	0	2	33	0.5	0.5	0%	6%	6%	0%	94%	94%
A pregnant woman experiencing bleeding requires urgent medical care	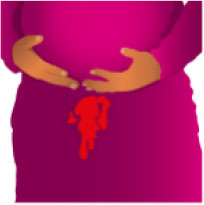	1	2	3	29	0	1	3%	9%	11%	6%	83%	89%
A woman with postpartum pain requires urgent medical care	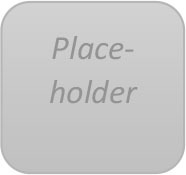	7	10	5	13	1.067	0.302	20%	14%	34%	29%	37%	66%
A pregnant woman showing multiple symptoms of pre-eclampsia requires urgent medical care	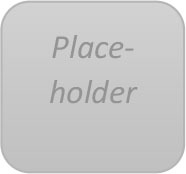	21	4	6	4	0.1	0.754	60%	17%	77%	11%	11%	23%

*The learning objective, pictogram representing the learning objective, McNemar categorization of paired responses to knowledge assessment, test statistics and significance, and desirable outcomes*.

* *indicates significance (p < 0.05)*.

**Table 3:** Summary of the neonatal health module.

**Neonatal health learning objectives**		**Pre and post responses** **(A = correct, B = incorrect)**				**McNemar statistics**	**Significance**	**Desired outcome** **(% of 35 question pairs)**			**Undesirable outcome** **(% of 35 question pairs)**		
	**Image**	**AA**	**AB**	**BA**	**BB**	***X*^**2**^**	***p***	**AA**	**BA**	**Combined**	**AB**	**BB**	**Combined**
A newborn with an eye infection, see health worker	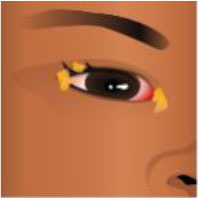	23	2	7	3	1.778	0.18	66%	20%	86%	6%	9%	14%
A newborn with a small skin infection, like an infected mosquito bite, see health worker	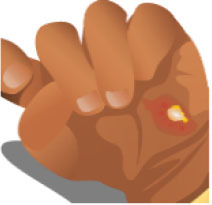	2	6	4	23	0.1	0.754	6%	11%	17%	17%	66%	83%
More than 6 loose watery stools in a day or bad smelling, see health worker	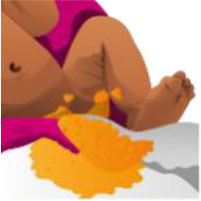	2	4	1	28	0.8	0.375	6%	3%	9%	11%	80%	91%
A newborn with fever, see health worker	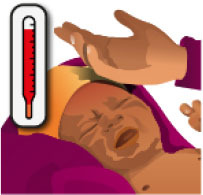	20	5	5	5	0	1	57%	14%	71%	14%	14%	29%
A newborn with many skin pustules, seek urgent medical care	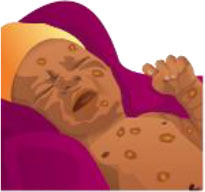	2	5	0	28	3.2	0.063	6%	0%	6%	14%	80%	94%
A non-responsive newborn, seek urgent medical care	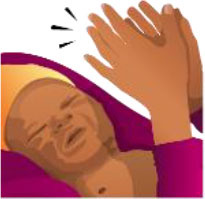	0	3	2	30	0	1	0%	6%	6%	9%	86%	94%
An umbilical infection is dangerous, seek urgent medical care	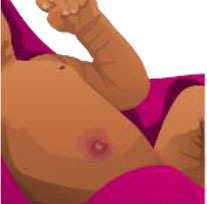	5	6	7	17	0	1	14%	20%	34%	17%	49%	66%
A newborn that is not feeding, seek urgent medical care	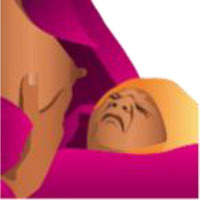	7	7	3	18	0.9	0.344	20%	9%	29%	20%	51%	71%
A low birth weight baby, seek urgent medical care at a hospital	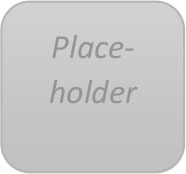	4	7	3	21	0.9	0.344	11%	9%	20%	20%	60%	80%

The learning objective, pictogram representing the learning objective, McNemar categorization of paired responses to knowledge assessment, test statistics and significance, and desirable outcomes. ^*^indicates significance (p < 0.05).

**Table 4:** Summary of the geohazard module.

**Geohazard learning objectives**		**Pre and post responses** **(A = correct, B = incorrect)**				**McNemar statistics**	**Significance**	**Desired outcome** **(% of 35 question pairs)**			**Undesirable outcome** **(% of 35 question pairs)**		
	**Image**	**AA**	**AB**	**BA**	**BB**	***X*^**2**^**	***p***	**AA**	**BA**	**Combined**	**AB**	**BB**	**Combined**
Narrow paths are low risk	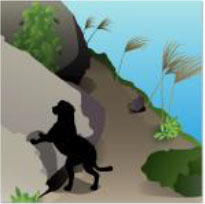	10	9	2	14	3.27	0.065	29%	6%	34%	26%	40%	66%
Evidence of rockfall is low risk	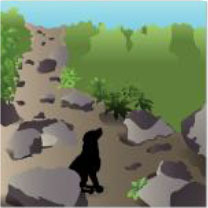	8	0	20	7	18.05	0.0000019^*^	23%	57%	80%	0%	20%	20%
Walking far from the edge of a cliff is low risk	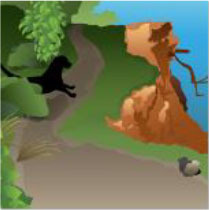	5	14	1	15	9.60	0.0009766^*^	14%	3%	17%	40%	43%	83%
Small cracks in the ground are low risk in sunny weather	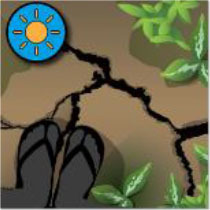	11	1	17	6	12.50	0.000145^*^	31%	49%	80%	3%	17%	20%
Shallow water is low risk in sunny weather	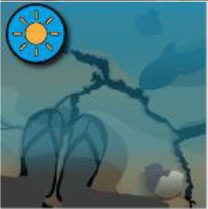	11	10	4	10	1.77	0.18	31%	11%	43%	29%	29%	57%
Large cracks in the ground are high risk	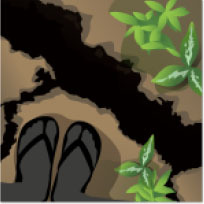	0	1	3	31	0.25	0.63	0%	9%	9%	3%	89%	91%
Small cracks in the ground are high risk in rainy weather	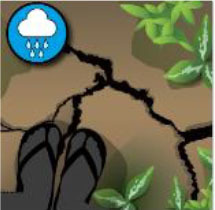	0	4	2	29	0.17	0.69	0%	6%	6%	11%	83%	94%
Walking near the edge of cliffs is high risk	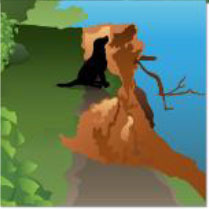	33	2	0	0	No Calc	No Calc	94%	0%	94%	6%	0%	6%
Shallow water is low risk in rainy weather	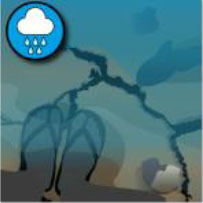	0	7	1	27	3.13	0.07	0%	3%	3%	20%	77%	97%

*The learning objective, pictogram representing the learning objective, McNemar categorization of paired responses to knowledge assessment, test statistics and significance, and desirable outcomes*.

* *indicates significance (p < 0.05)*.

Learning objectives with a high rate of AA response pairs suggests these pictograms were easy to interpret, and that most participants had the prior knowledge to respond correctly. In the maternal health module, these were a long labor and excessive breathlessness/tiredness, with 100% and 97% of responses coded AA, respectively. In the neonatal health module, AA responses were an infant's eye infection and an infant with many skin pustules at 86 and 71%, respectively. Among the geohazard learning objectives, walking near a cliff edge was easily interpreted as dangerous by participants, as 94% of response pairs were coded AA.

Learning objectives with a high rate of BB or AB coded responses suggest difficulties interpreting and judging the severity of conditions in pictograms, or simply a lack of knowledge of the condition. In the maternal health modules, these were nausea from morning sickness, postpartum hemorrhage, and limb prolapse during delivery.

Improvement from the pre-test to the post-test, coded BA, is of great interest because participants initially answered incorrectly, but interpreted and judged pictograms correctly in the post-test. Evidence of rockfalls and small cracks in the ground were successful learning objectives with relatively high numbers of responses coded BA, at 57 and 49%, respectively.

Analyzing learning objectives from each module as a set, the highest measured impact among the three modules is the geohazard module, with 16% of response pairs coded BA, whereas for maternal and neonatal the percentage of response pairs coded BA was 9 and 10% respectively. Several pictograms that had few desirable response pairs were described in focus group discussions as being difficult to interpret. Many participants did not recognize the blue color shading intended to depict water in streams or rivers, nor did they recognize black zig zag lines as cracks in the ground, so these images need further work and co-design. The highest improvement was found in the geohazards module, which achieved 41% desirable responses. This was the same overall percentage of desirable responses as the maternal health module, which scored highly due to higher prior knowledge of maternal health conditions. Prior knowledge in the geohazard module was low so a larger proportion of desirable responses were coded BA.

The learning objective of a pregnant woman with thick white discharge had a statistically significant negative change in knowledge (Table 2). When we discussed this condition in focus group discussions we found that one woman in the community with this condition had miscarried, so people judged it to be dangerous.

### Assessment of Knowledge Gain by Module and Overall

Statistical results of paired *t*-tests comparing individuals' pre-game test results with post-game test results are presented in Table 5. These give an indication of knowledge gained by playing the game. Mean and standard deviation of scores as well as and *T*-test results are divided by module. Scores are normalized to 100 for comparison.

**Table 5:** Knowledge assessment analysis by paired *T*-test of learning objective test scores (*n* = 35).

**Module**	**Knowledge assessment**	**Mean**	**Standard deviation**	**Standard error**	**T-statistics**	***p***
All (28 LOs)	Pre-game test	70	8.9	1.5	5.88	0.000001^*^
	Post-game test	78	7.7	1.3		
	Difference	7.7	7.7	1.3		
Maternal (10 LOs)	Pre-game test	77	13	2.2	2.89	0.0067^*^
	Post-game test	85	10	1.8		
	Difference	7.4	15	2.6		
Neonatal (9 LOs)	Pre-game test	69	11	1.8	2.63	0.013^*^
	Post-game test	75	12	2.1		
	Difference	6.0	14	2.3		
Geohazard (9 LOs)	Pre-game test	64	15	2.6	4.09	0.00025^*^
	Post-game test	73	13	2.1		
	Difference	9.5	14	2.3		

*Analysis is of all 28 learning objectives and divided into modules. Test scores are normalized to 100 and numbers are rounded to two significant figures except for T-statistics*.

* *indicates significance (p < 0.05)*.

Means of pre-game test scores indicate baseline or prior knowledge (Table 5). Participants had the most prior knowledge of maternal health and the lowest prior knowledge of geohazards with pre-game test means of 77 and 64, respectively.

Change in knowledge due to playing the mobile phone app is quantified by the difference, calculated as the normalized post-game test score minus normalized pre-game test scores. The average change in knowledge is greatest in the geohazard module (9.5), followed by maternal health (7.7 points), and then neonatal health (6.0 points). Average improvement of all 28 learning objectives is 7.7 points.

*T*-tests performed on all 28 learning objectives, presented in row “All,” show an improvement in scores for the entire intervention (*p* < 0.001). *T*-test results for each module; maternal health (*p* = 0.0067), geohazards (*p* < 0.001) and neonatal health (*p* = 0.013) modules results also showed statistically significant change in scores.

### Demographics and Phone Use

Characteristics of participants are summarized in Table 6. Smartphone ownership was of particular interest, and 54% of the 35 participants owned smartphones. Of these 19 smartphone owners, 8 were women and 9 were men.

**Table 6:** Summary of demographic information.

**Characteristic**	**Group**	***N***	**% of Total participants**
Smartphone ownership	Own smartphone	19	54%
	No smartphone	16	46%
Age	0–34 years	10	29%
	35+ years	25	71%
Education	0–4 years	12	34%
	5–9 years	6	17%
	10+ years	17	49%
Gender	Female	24	69%
	Male	11	31%
Community role	FCHV	19	54%
	Community women	5	14%
	Community men	11	31%
Village	Chyamrangbesi	17	49%
	Chandenimandan	11	31%
	Imadol	7	20%

### Stratified Results for Each Demographic

**Figure 5:**
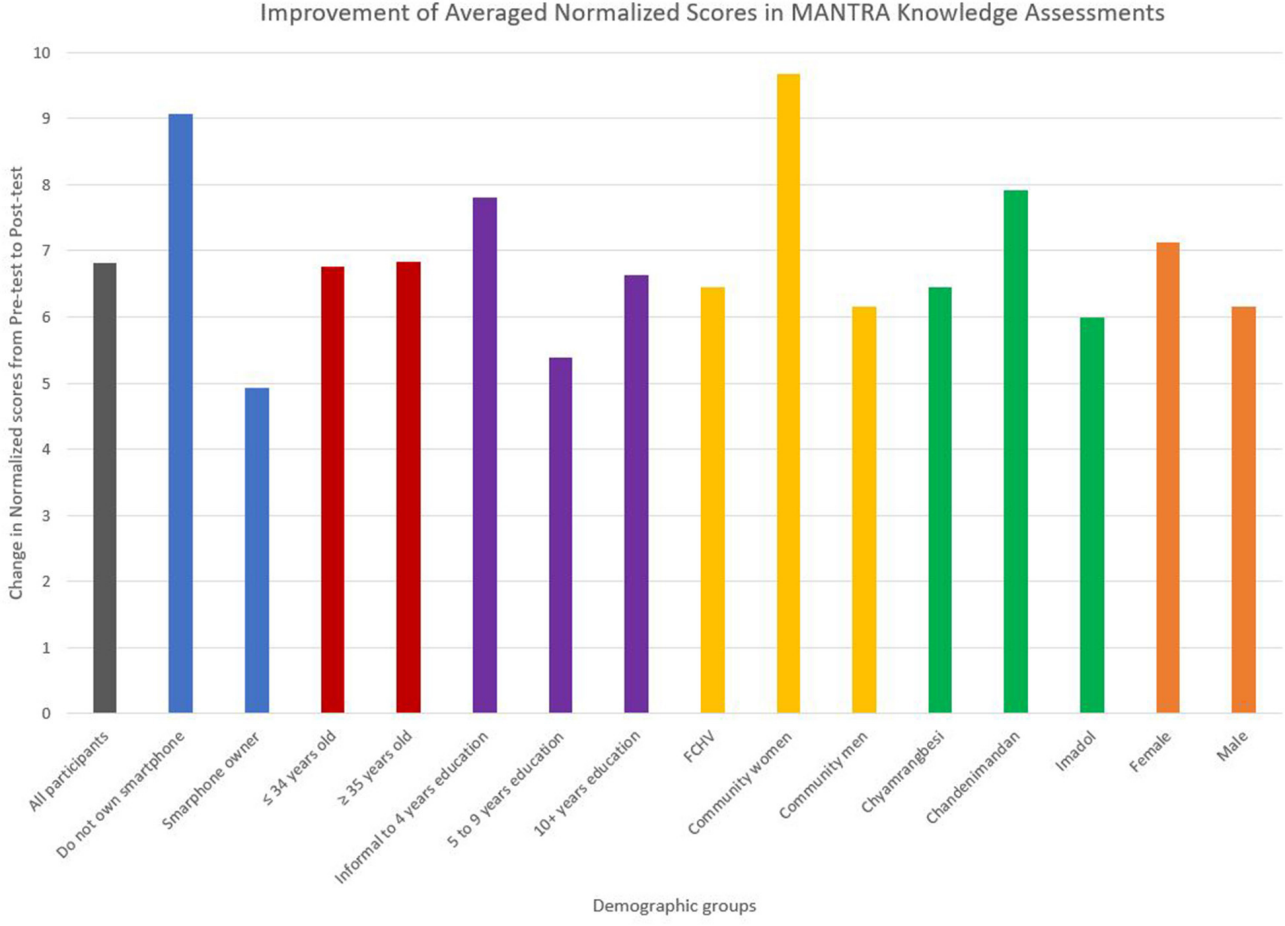
Change in knowledge from the pre-test to the post-test of the knowledge assessment from ANOVA statistical tests. Each color presents a demographic characteristic, and each bar within a color is the labeled group within that demographic characteristic. The average change in knowledge for the entire study sample population (*n* = 35) is included for visual comparison. This figure is to illustrate the similar change when the participant group (*n* = 35) was divided and compared based on demographic characteristics. The results are limited by the small sample size of the study and further division into demographic groups. Reprinted from (29).

The demographic information collected about our participants was used to compare the performance of different demographic groups on the paired *t*-test with ANOVA analyses. Figure 5 illustrates the change in knowledge for the demographic groups in Table 6. The averaged change in scores across the various demographic divisions fall within a narrow interval of 5 to 10 normalized points of improvement, roughly corresponding to 1 to 2 more correct answers in the post-test than the pre-test. The value of results from the ANOVA analyses are limited by the small sample size of the study. While being mindful of these limitations, the results suggest a similar improvement across demographic divisions from playing the game and supports repeating the ANOVA analyses in a larger future study.

### Key Insights From Observations and Focus Group Discussions

A vital insight from field test observations is that the participants naturally wanted to play together and assist each other. Discussion in focus groups highlighted that participants thought playing together would lead to transfer of knowledge through the community and assist with smartphone interactions [(35); Kayastha et al. in preparation].

## Discussion

### Results in Broad Context

Our results demonstrate that complex mHealth interventions designed for smartphones are plausible learning tools in the field. Comparisons of changes in mean score and transitions between incorrect to correct answers demonstrated which pictograms/learning objectives within the MANTRA serious game are effective and which ones are in need of improvement in the next iteration of the intervention. On the whole participants had more prior knowledge of the maternal health module learning objectives than those in the neonatal health and geohazard modules, likely because of their experience as FCHVs or as mothers. All modules had positive outcomes, but more of the geohazard respondents transitioned from incorrect to correct responses than in the other modules. Pictograms in need of revision to be more clearly understood included a person standing in water of a stream and black cracks in the ground depicting landslide risk in the geohazard module, and nausea, postpartum hemorrhage and limb prolapse in the maternal health module.

A common theme from focus group discussions [(35); Kayastha et al. in preparation] and observations is the tendency toward communal learning approaches. This transfer of knowledge between community members may increase engagement, impact, knowledge gain, and behavior change whilst also decreasing barriers to learning due to experienced smartphone users assisting inexperienced users [(35); Kayastha et al. in preparation].

### Limitations

Since we focused our data collection amongst FCHVs and reproductive aged women, for whom the game was devised, our sample is not representative of the general Nepalese population. Some potential participants from the broader community were unable to attend due to domestic responsibilities and travel times/distance. Possible sources of contamination are in the *in-situ* nature of the field sessions and participant expectations of the researchers. Since the game was specifically designed for Nepal, the artwork ought to be revised prior to deploying the serious game in a different setting. Our sample size is too small to be able to draw conclusions upon the responses of different population subgroups to the game. Despite this, we were able to evaluate the serious game on a small scale so as to support the next phase of the project at a larger scale. We were also able to contextualize the results of the statistical analyses by discussing the game in focus group discussions and by observing participants playing the game in a real-world setting, rather than in laboratory conditions. This approach allows researchers to “understand how technology is and can be used in the everyday real world, in order to gain new insights” regarding engagement, impacts, and behavior when faced with a new technology (36).

### Future Work

Future work should build on insights, challenges, and successes from this substudy and the overall MANTRA project. The next iteration of the MANTRA serious game should be tested on a larger sample, redesigning those learning objectives and pictograms which did not work well so as to improve knowledge gain.

Future field evaluations will aim to cover a more representative larger population by incorporating individuals across generations and genders randomly selected from various regions in Nepal. Actions to improve knowledge gained through the intervention include redesigning pictograms, delivering more non-textual information through animation and audio, and providing an instruction module in the game. We might also work through FCHVs to educate players before playing the game. Measuring knowledge gain in the game itself rather than through paper tests would be ideal for the next evaluation of the serious game. A larger sample of participants would improve data quality and enable more robust statistical comparisons of population subgroups.

Communication of the learning objectives that were not successful in this development phase will be improved through co-creative iterative processes and by exploring the addition of animation, audio, or both to clarify and convey complex conditions. A potential pathway for scale up or an intermediate step is training FCHVs to incorporate the serious game into existing community workshop programs.

Studies like MANTRA contribute to the growing evidence base supporting serious games as a delivery method for educational messages in mHealth and beyond. Further research is worthwhile to determine best practice and effective designs to maximize learning in health education and decision-making, as well as motivation and engagement. Following the insights of this MANTRA study, future interventions in Nepal and other LMICs may consider the capabilities and advantages of visual designs over text-based designs in societies with low literacy.

## Conclusions

The MANTRA study developed a set of learning objectives represented as pictograms for the serious game across three modules: maternal health, neonatal health and geohazards. Using simple image matching with a drag and drop interface, users practiced decision-making for a range of maternal and neonatal health conditions and geohazard conditions.

Analysis of test results provides encouraging evidence that participants gained knowledge from playing the serious game on smartphones. Statistical analyses of the knowledge gain assessments demonstrated a positive change in test scores between pre- and post-game test scores and showed by learning objective the proportion of desirable or undesirable responses.

Our MANTRA study demonstrates the benefit of interdisciplinary collaboration to develop educational content combining maternal health, neonatal health, and geohazards. During the 2015 Nepal earthquake, pregnant and perinatal women faced major challenges and disruptions to their healthcare. Development of a smartphone serious game to provide information about how to respond to maternal and neonatal health problems and geohazards is a useful supplement to existing rural health infrastructure. Targeting a largely illiterate population with such a serious game is an entirely novel agenda with promising educational impact.

## Data Availability Statement

The raw data supporting the conclusions of this article will be made available by the authors, without undue reservation.

## Ethics Statement

The studies involving human participants were reviewed and approved by University College London Ethics Committee in London, United Kingdom [10547/001] and the Nepal Health Research Council in Kathmandu, Nepal [Reg. No. 105/2017]. The patients/participants provided their written informed consent to participate in this study.

## Author Contributions

SM contributed to design of the work, drafting the article, and conducting the statistical analyses. DS contributed to design of the work and developed the pictograms. AB contributed to design of the work. NS contributed to the conception and design of the work, particularly perinatal health messages, and as well as acquisition of field data. AA contributed to the acquisition of field data. SB and MF contributed to conception of the work. AA, SB, and NS assisted to contextualize the content of the game. GH contributed to conception, design of the work, and particularly the geohazard messages. RK interpreted focus group data. PK contributed to conception, design, analysis, interpretation, and drafting of the work. All authors read and approved the manuscript.

## Conflict of Interest

GH was employed by the company Hearn Geoserve, Ltd. The remaining authors declare that the research was conducted in the absence of any commercial or financial relationships that could be construed as a potential conflict of interest.
